# Microbiota and Probiotics: The Role of Limosilactobacillus Reuteri in Diverticulitis

**DOI:** 10.3390/medicina57080802

**Published:** 2021-08-05

**Authors:** Andrea Piccioni, Laura Franza, Vanessa Vaccaro, Angela Saviano, Christian Zanza, Marcello Candelli, Marcello Covino, Francesco Franceschi, Veronica Ojetti

**Affiliations:** 1 Emergency Department, Fondazione Policlinico Universitario A. Gemelli, IRCCS, 00168 Roma, Italy; marcello.candelli@policlinicogemelli.it (M.C.); marcello.covino@policlinicogemelli.it (M.C.); francesco.franceschi@policlinicogemelli.it (F.F.); 2 Emergency Department, Università Cattolica del Sacro Cuore, 00168 Roma, Italy; cliodnaghfranza@yahoo.it (L.F.); vanessa.vaccaro@libero.it (V.V.); saviange@libero.it (A.S.); christian.zanza@live.it (C.Z.); veronica.ojetti@policlinicogemelli.it (V.O.)

**Keywords:** diverticulitis, Lactobacillus, acute diverticolitis, *Lactobacillus reuteri*, immune system, inflammatory diseases, microbiota, probiotic

## Abstract

The microbiota is the set of commensal microorganisms, residing in the organism, helping proper functioning of organs and systems. The role that the microbiota plays in maintaining the health of vertebrates is widely accepted, particularly in the gastrointestinal system, where it is fundamental for immunity, development, and conversion of nutrients. Dysbiosis is an alteration of the microbiota which refers to a disturbed balance, which can cause a number of pathologies. Probiotics have proven to be effective in modulating the microbiota of the gastrointestinal system and, therefore, in promoting the health of the individual. In particular, Lactobacilli are a group of Gram-positive bacteria, which are able to produce lactic acid through glucose metabolism. They are present in different microenvironments, ranging from the vagina, to the mouth, to different tracts of the small intestine. In the present review, we will discuss the use of *Limosilactobacillus* in human health in general and more specifically in diverticulitis. In particular we analyze the role of *Limosilactobacillus reuteri* and its anti-inflammatory action. For this review, articles were identified using the electronic PubMed database through a comprehensive search, conducted by combining key terms such as “diverticulitis”, “*Limosilactobacillus reuteri*”, “human health and disease”, “probiotics”. We selected all the articles published in the last 10 years and screened 1017 papers. Articles referenced in the screened papers were evaluated if considered interesting for our topic. Probiotics have proven to be effective in modulating the microbiota of the gastrointestinal system and, therefore, in promoting the health of the individual. The importance of probiotics in treating diverticular disease and acute diverticulitis can be further understood if taking into consideration some pathophysiological aspects, associated to the microbiota. *L. reuteri* plays an important role in human health and disease. The effectiveness of *L. reuteri* in stimulating a correct bowl motility partly explains its effectiveness in treating diverticulitis. The most important action of *L. reuteri* is probably its immunomodulating activity. Levels of IL-6, IL-8, and Tumor necrosis factor (TNF-alpha) are reduced after supplementation with different strands of Lactobacilli, while T-regulatory cells increase in number and activity. Anyway, new mechanisms of action of probiotics come to light from the many investigations currently taking place in numerous centres around the world and to improve how exactly probiotic administration could make the difference in the management of diverticular disease and acute diverticulitis.

## 1. Introduction

The microbiota is the set of commensal microorganisms, residing in the organism, helping proper functioning of organs and systems. The role that the microbiota plays in maintaining the health of vertebrates is widely accepted, particularly in gastrointestinal system, where it is fundamental for immunity, development, and conversion of nutrients. Dybiosis is an alteration of the microbiota which refers to a disturbed balance, which can cause a number of pathologies. Probiotics have proven to be effective in modulating the microbiota of the gastrointestinal system and, therefore, in promoting the health of the individual.

In particular, Lactobacilli are a group of Gram-positive bacteria, which are able to lactic acid through glucose metabolism. They are present in different microenvironments, ranging from the vagina, to the mouth, to different tracts of the small intestine.

In the present review, we will discuss the use of Limosilactobacillus in human health in general and more specifically in diverticulitis. In particular, we analyse the role of *Limosilactobacillus reuteri* and its anti-inflammatory action.

## 2. Lactobacilli as Probiotics: Which Ones and Where?

The microbiota is the set of commensal microorganisms, residing in the organism, helping proper functioning of organs and systems [1]. The role that the microbiota plays in maintaining the health of vertebrates is widely accepted, particularly in gastrointestinal system, where it is fundamental for immunity, development, and conversion of nutrients [2].

Dybiosis is an alteration of the microbiota which refers to a disturbed balance, which can cause a number of pathologies [2].

Probiotics have proven to be effective in modulating the microbiota of the gastrointestinal system and, therefore, in promoting the health of the individual [3].

Probiotics are defined by the World Health Organization as “live microorganisms which, if administered in the right amount, benefit the host”; to be considered effective, a probiotic must have specific requirements and characteristics: it must have the ability to survive in the gastrointestinal tract, adhere to the epithelium of the mucosa, be resistant to gastric acids, and must be free of transferable genes of antibiotic resistance [4]. Indeed, there is evidence that some species of Lactobacilli, if not used correctly, can determine severe diseases [5]. Lactobacilli are a group of Gram-positive bacteria, which are able to produce lactic acid through glucose metabolism. They are present in different microenvironments, ranging from the vagina [6], to the mouth [7], to different tracts of the small intestine [8].

There are about 170 different species of Lactobacilli, which play an important role in different contexts [9] but are often difficult to differentiate from one another [5]. For instance, in the vaginal microbiota, *Lactobacillus crispatus, Lactobacillus gasseri, Lactobacillus jensenii and Lactobacillus iners* are the most common found species, while they are not as common in other microenvironments [10]. Additionally, in the case of probiotics, some species are more effective than others.

*Lactobacillus acidophilus*, *Lactobacillus casei, Lactobacillus brevis, Lactobacillus fermentum, Lactobacillus parabuchneri, Lactobacillus bulgaricus, Lactobacillus rhamnosus* GG are the most commonly used species, particularly for commercial purposes, both in producing probiotics and in fermentation of different types of food [5]. All these species have proven effective in treating different types of diseases. Lactobacillus acidophilus can be used in traveller’s diarrhoea, Lactobacillus casei in constipation and rheumatoid arthritis, Lactobacillus brevis in biliary disorders, Lactobacillus fermentum in vaginosis and in Staphylococcus infections, Lactobacillus bulgaricus improves immune status, Lactobacillus rhamnosus GG also improves immune status, particularly in viral infections [11].

While not as commonly used as other species, *Limosilactobacillus* is also effective in different contexts, such as bacteraemia, gastroenteritis, colitis, hospitalizations, particularly in infants [11].

In Table 1, we present a short summary of the different species of Lactobacilli, with implications on human health.

## 3. Limosilactobacillus Reuteri in Human Health and Disease

*Lactobacilli spp.* are the most used probiotics and one of the species most frequently found in food products [33]. As discussed above, numerous species belong to this genus including *L. acidophilus, L. rhamnosus, L. bulgaricus, L. casei*, and *L. reuteri*. *L. reuteri* possesses all the requisites to be considered an effective probiotic [2]. It has proven to be effective in influencing the diversity, composition, and metabolic function of the microbiota, not only in the intestine but also in the oral and vaginal area [34,35].

The antimicrobial and immunomodulating power of *L. reuteri* lies in the metabolites it can produce [2]. Among these, one of the most important is reuterin, a molecule consisting of a mixture of three hydroxypropionaldehyde 3 hpa, which in turn breaks down spontaneously into acrolein or a cytotoxic electrophile, capable of inhibiting a large amount of gram-negative bacteria [2,36].

Other metabolites that make *L. reuteri* effective against many types of gastrointestinal infections are acetic acid, ethanol and lactic acid [37,38] 1. Another metabolite that confers immunomodulating properties in the gastrointestinal tract to *L. reuteri* is histamine [2].

Some strains of *L. reuteri* are also able to convert the amino acid L-histidine, of dietary origin, into biogenic histamine. Some studies have shown that histamine produced by *L. reuteri* can suppress the production of tumour necrosis factor (TNF), produced by stimulated human monocytes [39]. The clusters of chromosomal genes responsible for these properties are histidine decarboxylase A-B and C. The research has also found that oral administration of hdc + *L. reuteri* could effectively suppress intestinal inflammation in a mouse colitis model [39].

*L. reuteri* is also able to produce lipopolysaccharide (LPS), which is extremely important for the adhesion of *L. reuteri* to intestinal cells and the formation of a biofilm [40]. The formation of biofilm is an important feature for *L. reuteri,* because it allows it to survive in the intestinal environment and to protect the epithelium from the adhesion of other pathogenic microorganisms through both steric hindrance and competitive inhibition and by triggering the immune response of the host [41]. It has also been shown that LPS produced by *L. reuteri* can avoid the adhesion of *E. coli* to intestinal epithelial cells in pigs in vitro and also suppresses the expression of some *E. coli*-induced pro-inflammatory cytokines such as interleukin (IL)-6 and IL-1b [42].

The tryptophan catabolites have been recognized as ligands for the aryl hydrocarbon receptor, whose activation induces the expression of IL-22 and other cytokines by innate lymphoid cells. In addition, tryptophan derivatives can induce the development of double-positive regulatory CD4+ and CD8+ intraepithelial lymphocytes in anaryl hydrocarbon receptor (AhR)-dependent manner [43].

One of the infections in which *L. reuteri* has proved effective in eradicating and reducing symptoms is chronic *H. pylori* infection [2], one of the most frequent chronic infections and one of the main causes of peptic ulcers, as well as a risk factor for developing some types of gastric cancer. *L. reuteri* acts by competing with *H. pylori* thus, inhibiting its adhesion to glycosidic receptors [44]. This mechanism of action is very effective in eradicating the infection and in reducing symptoms, while avoiding the side effects associated with antibiotic therapies [45].

The integrity of the intestinal barrier is essential to prevent the entry of pathogens and external toxins. When it is disturbed, the intestine becomes permeable allowing the passage of antigens and toxins into the intestinal lumen thus, triggering a pathological process. Probiotics, in particular *L. reuteri*, are very effective in ensuring the integrity of the barrier itself [46].

A study carried out on mice has shown that the administration of a mixture of Lactobacilli, including *L. reuteri*, can increase the expression of tight junction proteins in intestinal epithelial cells. This finding has been further confirmed by studies carried out on pigs [38,47].

The ability to maintain the intestinal barrier permeability of *L. reuteri* has also been demonstrated in humans. In children with atopic dermatitis, for instance, in which the increase in intestinal permeability was related to the pathogenesis of the disease [48], the administration of *L. reuteri* reduced intestinal permeability, as demonstrated by the reduction of the lactulose/mannitol ratio, and associated symptoms [49].

**Figure 1:**
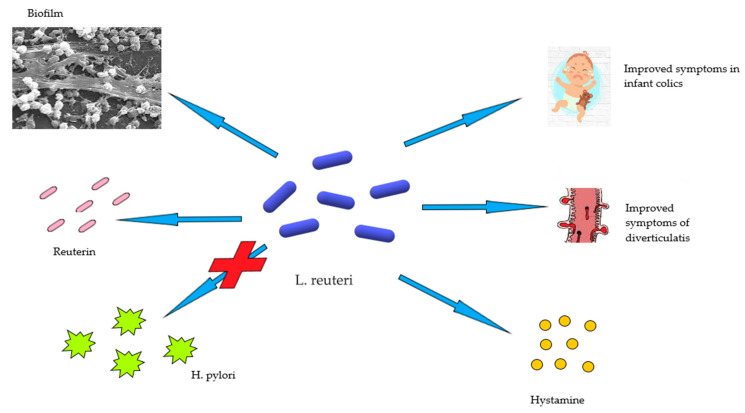
The role of L. reuteri in human health and disease.

*L. Reuteri* has also been proven effective in disorders of the early stages of life such as colic or disorders characterized by excessive crying, which affects 10–30% of newborns. In a study conducted by Guolin et al., breastfed infants younger than 4 months of age were recruited and divided into two groups, a placebo and a group to which *L. reuteri* was administered. At the end of the 4 weeks supplementation, it was found that 100% of the children treated with *L. reuteri* had a reduction in mean crying time and a decrease in maternal depression as a secondary effect. The same effects were found in only the 15, 7% of children treated with placebo, suggesting that *L. reuteri* may be a viable option for the treatment of infantile colic [49] (Figure 1).

## 4. Acute Diverticulitis

Colon diverticula are blind-ended extroversions involving the mucosal and submucosal layer of the intestinal wall [50]. They represent one of the most common pathologies in inpatient and outpatient patients [51] and the most frequently encountered finding in colonoscopies [52]. They can occur anywhere in the large intestine and their distribution depends heavily on the country of origin. In the industrialized Western world, diverticula strains are much more frequent at the sigmoid level. They can be single but are frequently fund as multiple [53].

The prevalence of diverticula is very similar in men and women [51] and it is often directly proportional to the patient’s age [54]. Studies have estimated an increase in diverticula occurring in about half of people between 60 and 80 years old, up to 70% in people aged 80 [55,56].

To date, the process driving the pathogenesis is still not fully understood; however, it is clear that it lies within the interaction of various risk factors determining an increase in intraluminal pressure and causing herniation of the intestinal wall, where the rectus vessels penetrate the colon wall [57].

The risk factors are represented by age and genetics, as demonstrated by the incidence of the disease among homozygote twins, when compared to heterozygotes [58]. Poor colon wall motility, low-fibre diet, obesity, smoking, consumption of red meat, constipation, alterations of the intestinal microbiota, use of some drugs in particular non-steroidal anti-inflammatory drugs, aspirin, opioids, and comorbidities are also important factors in the development of the disease. In the literature, it is possible to find various associations between the development of diverticula and other diseases, in particular diabetes and hypertension [55,59].

Diverticula can be asymptomatic for life or can become inflamed and symptomatic [57]. However, recent studies have estimated that less than 5% of patients with diverticulosis will become symptomatic [60].

Diverticulitis can be divided into two categories: simple and complicated [59]. Simple diverticulitis is characterized by the inflammation of one or more adjacent diverticula and the pericolic tissue [61]. The complicated form, which occur in 12% of cases, is represented primarily by an abscess followed by perforation, obstruction, and fistulisation [59].

Simple diverticulitis is also known to cause pain, generally localized to the left lower quadrant and may be accompanied by nausea, vomiting, alterations of the alvo [59]. Occult blood in the stool is particularly rare, while hemodynamic instability and abdominal rigidity are findings compatible with the complications, such as free perforation and/or generalized peritonitis [62].

Since diverticula are asymptomatic in most cases, they are often an occasional finding during a colonoscopy performed for other reasons [55].

Abdominal computed tomography scan (CT) scan is used in case of acute symptoms, when colonoscopy is not indicated [55]. An alternative to CT can be abdominal ultrasound, with a sensitivity and specificity of about 90%. Magnetic MRI can also be used in suspected diverticulitis in patients for whom CT or ultrasound are not indicated. X-ray can be used in suspicion of abdominal perforation or to exclude other causes of abdominal pain such as intestinal obstruction. However, this methodology cannot be used to diagnose diverticulitis and/or abscess [59].

Uncomplicated acute diverticulitis can be treated directly in the hospital facilities and includes a treatment involving a liquid diet and use of antibiotics, in particular a combination of metronidazole and ciprofloxacin.

Hospitalization is indicated in all those patients that are unable to take oral therapy, are suffering from severe comorbidities, or do not present any improvement despite oral therapy or face complications. Clinical improvement usually occurs within 34 days of onset of symptoms [57].

The role of surgery has changed through time: abscesses are treated with antibiotic therapy or percutaneous drainage while the use of surgery is reserved for cases of peritonitis. Besides, the associated comorbidities and the recurrence of episodes of inflammation are the factors to be considered when evaluating the surgical approach [62].

There is no consensus on the optimal therapy to be taken as secondary prevention. Although, a diet with a high fibre content is recommended, but also the use of rifaximin to be taken in cycles [57].

Studies have also shown that the use of mesalazine is not superior to placebo in reducing the likelihood of relapse but reduces abdominal symptoms [63].

## 5. Microbiota and Acute Diverticulitis

As discussed above, diverticulitis is defined as an inflammation of a herniation of colonic mucosa and submucosa through the muscle layer. Inflammation is caused in particular by local factors, including the microbiota, but it is not completely clear whether or not the microbiota could play a role in determining the herniation in the first place [64].

Dietary factors are key in determining the onset of diverticular disorder and they can also change the composition of one’s microbiota. It is not possible to say without doubt whether the changes in the composition of the microbiota may act as an enhancing factor in the development of diverticulitis, but it is worth nothing that the microbial species associated to diverticular disease are Enterobacteriaceae, Streptococcus and Bacteroides, while so called “good bacteria” (e.g., *Bifidobacteria* and *Lactobacilli*) are reduced [65].

The role of microbiota in determining diverticular inflammation is instead more straightforward. Recurring diverticulitis not susceptible to surgery has been treated in a successful and lasting way with faecal transplant, [66]. Further confirmation of the role microbiota plays in the development and progression of diverticular disease, is that patients receiving faecal transplant for *C. difficile* infection and who also presented mild forms of diverticular disease, developed after the procedure their first episode of diverticulitis [67].

A reduction in taxa with anti-inflammatory activity, such as Clostridium cluster IV, Lactobacilli and Bacteroides are all changes that have been observed in patients who were about to manifest acute diverticulitis. At the same time, also an overgrowth of Bifidobacteria, Enterobacteriaceae and Akkermansia have been reported [64]. Additionally, an increase in Proteobacteria is quite typical, and these changes could be used to diagnose the disease in its earlier stages [68].

Finally, the importance of the microbiota in diverticular disease has been demonstrated indirectly by the therapies used to treat it. Rifaximin and probiotics have proven to be effective, at least in clinical trials.

Rifaximin has been used to treat Symptomatic Uncomplicated Diverticular Disease in a clinical trial and results were encouraging, in terms of symptom control. Interestingly, the changes in clinical presentation, also corresponded to changes in faecal microbiota composition, with a reduction of *Roseburia, Veillonella, Streptococcus* and *Haemophilus* [69]. In another study, also *Akkermansia* resulted reduced [70].

Lactobacilli have demonstrated to reduce Symptomatic Uncomplicated Diverticular Disease, with a reduction of bloating and abdominal pain [71], while *Lactobacillus salivarius, Lactobacillus acidophilus* and *Bifidobacterium lactis* have proven effective in the treatment of acute diverticulitis [72].

## 6. Limosilactobacillus and Diverticulitis

As discussed above *L. reuteri* plays an important role in human health and disease. Its effects on gut health are quite interesting, studied in particular in terms of fighting chronic constipation. Given its relatively safe profile, supplementation has been used to treat chronic constipation in children, with encouraging results [73].

The effectiveness of *L. reuteri* in stimulating a correct bowl motility partly explains its effectiveness in treating diverticulitis.

While data on this particular topic are not abundant, one study in particular, by Petruziello et al. [32], has highlighted that supplementation with this particular strand significantly reduced symptoms in acute uncomplicated diverticulitis. Interestingly, also inflammatory markers were reduced, further reinforcing the results of the study.

The mechanisms through which these positive responses take place are different: while on the one hand an action on intestinal motility does take place and is important, it is worth noting that the most important action of *L. reuteri* is probably its immunomodulating activity. Levels of IL-6, IL-8, and TNF-alpha are reduced after supplementation with different strands of *Lactobacilli*, while T-regulatory cells increase in number and activity [74].

Additionally, there appears to be a higher expression of tight junction proteins claudin-1, occludin, and zonulin-1.

The described actions are likely to be at least in part modulated by the increased production of butyrate, induced by this strand of bacteria, including *L. reuteri*.

While they do not directly produce butyrate, they are capable of activating it [75].

The effects of these supplements strongly depend on the host’s immunity, particularly on T-regulatory cells, even though it has been suggested by recent studies that *L. reuteri* might be capable of modulating the activity of this compartment of immunity. Liu et al. have observed in murine models that, while other strains of *Lactobacilli* are not as effective in reducing inflammation if T-regulatory cells are deficient, *L. reuteri* might be still capable of modulating inflammation and microbiota, which suggests there are other mechanisms underlying its action [76].

## 7. Probiotics in Diverticulitis: Mechanisms of Action

The importance of probiotics in treating diverticular disease and acute diverticulitis can be further understood if taking into consideration some pathophysiological aspects, associated to the microbiota. Changes in microbiota composition have been associated to altered nervous response in the gut, which leads to neuronal and muscular dysfunction, and eventually to abdominal symptoms [77].

Additionally, a decreased presence of anti-inflammatory bacterial species might be linked to mucosal inflammation, resulting in a vitious circle, in which dysbiosis and inflammation promote each other [78].

Dysbiosis and mucosal inflammation can also lead to dysmotility, further promoting bacterial translocation from the lumen of the diverticulum to perivisceral area. In such way, it is possible for Toll-like receptors of innate immunity to be stimulated and activated, with a subsequent inflammatory reaction at the level of perivisceral tissues [79].

This evidence has led researchers to consider changes in peri-diverticular bacterial flora as a critical element in acute diverticulitis pathogenesis, in a similar way to acute appendicitis. Overall, stasis of faecal material within diverticula can be favoured by a prolonged colonic transit, which promotes an altered microflora and bacterial overgrowth. Mucosal barrier function can be then impaired, determining an inflammatory reaction, through of cytokine release; a low-grade, localized microscopic colitis can take place, evolving towards microperforation and acute diverticulitis [80].

It appears clear that microbial colonization plays an important role in at least promoting diverticular disease, thus changing its composition through probiotic becomes an interesting therapeutic strategy. Interestingly, use of probiotics also results in direct changes in inflammatory patterns, as reported by Quigley. Indeed, probiotics have the ability to modify localized and persistent inflammation which is constantly present in some patients suffering from acute diverticulitis, even between bouts of the disease, which may also sustain symptoms’ development in individuals affected by uncomplicated diverticular disease [81]. Foligne and colleagues [82] have studied, in the context of IBD, thirteen strains of probiotics in terms of anti-inflammatory properties and, among these, *L. acidophilus* and *L. salivarius* Ls33 seemed to be the best-performing, in terms of increased induction of IL-10 and decreased induction of IL-12.

Data from in vitro and in vivo studies concerning *L. salivarius* Ls33 suggest that it is linked to an improved recovery of tissue inflammation in a rat colitis model [83].

The intestinal bacterial flora also produces outer membrane vesicles, which play an important role in microbiota–host communication, through the action of adhesins, sulfatases and proteases and pathways such as micropinocytosis, clathrin- and lipid raft-dependent endocytosis [84].

These outer membrane vesicles positively impact mucosal immunity and its signaling pathways. While this could be an interesting further explanation of the mechanisms underlying the effectiveness in treating diverticulitis and abdominal disorders with probiotics, it is still not completely understood and data are still lacking [85,86].

Brandimarte et al. [87] have discussed the pros and cons, regarding evidence on probiotic action in diverticular disease. Overgrowth and alteration of gut microbiota play an important role in the development of inflammation, which is key to diverticular disease development, thus there is a clear rationale for the use of probiotics, aiming to restore a healthy microenvironment in the colon. The involved mechanisms, range from bacterial translocation, inflammation, competitive inhibition of pathogenic bacteria, immune modulation and are all potentially a target of probiotic therapy [88,89].

## 8. Methods

For this review, articles were identified using the electronic PubMed database through a comprehensive search, conducted by combining key terms such as “diverticulitis”, Limosilactobacillus Reuteri”, “human health and disease”, “probiotics”.

English-language articles were screened for relevance. Full review of publications for the relevant studies was conducted, including additional publications that were identified from individual article reference lists. At first, the literature search was individually conducted by the single authors, who subsequently confronted each other in order to include in the review only the most recent and most relevant articles.

We selected all the articles published in the last 10 years and screened 1017 papers between March and June 2021. Articles referenced in the screened papers were evaluated if considered interesting for our topic.

## 9. Conclusions and Discussion

Probiotics have proven to be effective in modulating the microbiota of the gastrointestinal system and, therefore, in promoting the health of the individual.

The importance of probiotics in treating diverticular disease and acute diverticulitis can be further understood if taking into consideration some pathophysiological aspects, associated to the microbiota.

Changes in microbiota composition have been associated to altered nervous response in the gut, which lead to neuronal and muscular dysfunction, and eventually to abdominal symptoms.

Additionally, a decreased presence of anti-inflammatory bacterial species might be linked to mucosal inflammation, resulting in a vitious circle, in which dysbiosis and inflammation promote each other.

In the present review, we will discuss the use of *Limosilactobacillus* in human health in general and more specifically in diverticulitis.

In particular, the effectiveness of *L. reuteri* in stimulating a correct bowl motility partly explains its effectiveness in treating diverticulitis.

L. Reutery significantly reduced symptoms in acute uncomplicated diverticulitis and it is shown by the reduced levels of inflammatory markers.

The mechanisms is probably its immunomodulating activity. Levels of IL-6, IL-8, and TNF-alpha are reduced after supplementation with different strands of *Lactobacilli*, while T-regulatory cells increase in number and activity.

Yet, the data present on this matter are not enough to find robust conclusions on the efficacy of probiotics in diverticular disease, as confirmed by a recent expert consensus with a submaximal level of agreement [90].

Overall, there still is no standard protocol in terms of probiotic use in diverticular disease, given that the data we have do not come from large studies and there still is a lack of robust information [87].

New mechanisms of action for probiotics have come to light from the many investigations currently taking place in numerous centres around the world.

New protocols should be established in order to study how exactly probiotic administration could make the difference in the management of diverticular disease and acute diverticulitis.

## Figures and Tables

**Table 1:** Lactobacilli and their effect on human health.

Species	Application	Reference
*Lactobacillus acidophilus*	Reduction of cholesterol; reduced in type 2 diabetes; improvement of Irritable bowel syndrome (IBS) symptoms; reduced inflammation.	[12,13,14,15,16]
*Lactobacillus casei*	Immune modulation; improvement of Chron’s disease and rheumatoid arthritis symptoms; alleviation of constipation.	[17,18,19]
*Lactobacillus brevis*	Improved gut barrier function; oxidative stress reduction.	[20,21]
*Lactobacillus fermentum*	Symptomatic colitis improvement; protective against *Staphylococcus* infection; improved response to viral infections.	[22,23,24]
*Lactobacillus bulgaricus*	Immune modulation; improved humoral response: promotion of wound healing	[25,26,27]
*Lactobacillus rhamnosus* GG	Improves symptoms of gastroenteritis; reduction of antibiotic resistance.	[28,29]
*Limosilactobacillus reuteri*	Improves diarrhoea; reduced symptoms of uncomplicated diverticulitis; metabolic modulation	[30,31,32]

## Data Availability

Not applicable.
